# A180 PLACEBO RATES IN MICROSCOPIC COLITIS RANDOMIZED TRIALS: APPLICATIONS FOR FUTURE DRUG DEVELOPMENT USING A HISTORICAL CONTROL ARM

**DOI:** 10.1093/jcag/gwac036.180

**Published:** 2023-03-07

**Authors:** P G Hamilton, K Buhler, G Kaplan, C Lu, C Seow, K Novak, R Panaccione, C Ma

**Affiliations:** 1 Internal Medicine Residency Program, Cumming School of Medicine - University of Calgary; 2 University of Calgary, Calgary , Canada; 3 Gastroenterology and Hepatology, University of Calgary, Calgary , Canada

## Abstract

**Background:**

There remains a need to develop effective medical therapies for patients with microscopic colitis (MC) who do not respond, are intolerant, or relapse on budesonide. Conducting randomized trials in MC is logistically and ethically challenging: budesonide is highly effective, and therefore, some institutional review boards have not allowed trials that randomize MC patients to placebo. However, comparing an investigational drug to budesonide is statistically infeasible: powering a non-inferiority study against a budesonide comparator arm with 90% power for a 10% non-inferiority margin would require over 700 subjects, yet fewer than 400 patients have been randomized in all historical MC trials. Therefore, alternative trial designs should be explored in MC, including the use of a historical control arm.

**Purpose:**

To conduct a systematic review and meta-analysis to determine the proportion of placebo responders in MC trials that will inform future trials using a historical placebo comparator, and evaluate factors associated with placebo response.

**Method:**

EMBASE, MEDLINE, and CENTRAL were searched from inception to January 7, 2022, and supplemented with conference abstracts to identify randomized controlled trials (RCTs) using a placebo comparator in adult patients with confirmed MC (either lymphocytic, collagenous, or mixed populations but excluding incomplete MC). The proportion of clinical and histologic responders in the placebo arms were pooled using random-effect models, statistical heterogeneity was evaluated using the I2 method, and the Freeman-Tukey double arcsine transformation was used to compute 95% confidence intervals (CI) using the score statistic and exact binomial method. All analyses were conducted in Stata 17.0.

**Result(s):**

Twelve placebo controlled RCTs were included, evaluating a total of 391 patients (163 randomized to placebo). The pooled placebo clinical response rate was 24.4% [95% CI 12.4%, 38.4%] (Figure 1), with substantial heterogeneity (I2=60.8%, p<0.01). The pooled histologic response rate was 19.9% [95% CI: 5.3%, 39.0%], with substantial heterogeneity (I2=66.4%, p<0.01). Subgroup analysis demonstrated higher placebo responses in lymphocytic colitis (39.9% [95% CI: 23.9%, 56.7%]) compared to collagenous colitis (19.8% [95% CI: 5.9%, 37.8%]), but not by allowance of baseline anti-diarrheals. Leave-one-out meta-analysis showed a reduction in heterogeneity after removal of Miehlke et al. 2014 (placebo response 21.0% [95% CI: 11.5%, 32.1%], I2=28.6%, p=0.17).

**Image:**

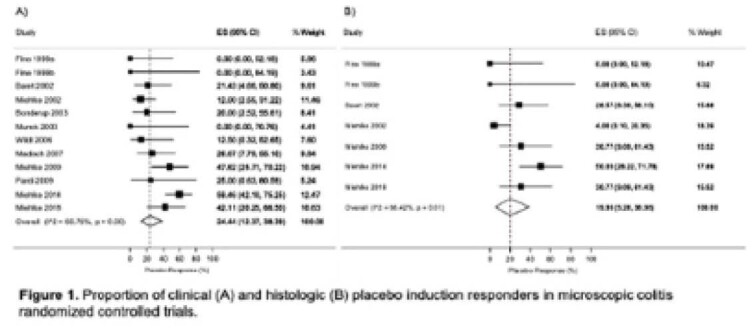

**Conclusion(s):**

Approximately 1 in 4 patients in MC trials will respond clinically to placebo and 1 in 5 will demonstrate a histologic response, although with substantial heterogeneity. T his highlights the need for standardized outcome definitions in MC trials and can serve to inform a Bayesian prior estimate for future trials that may consider using a historical placebo comparator.

**Please acknowledge all funding agencies by checking the applicable boxes below:**

None

**Disclosure of Interest:**

P. Hamilton: None Declared, K. Buhler: None Declared, G. Kaplan Grant / Research support from: Ferring, Janssen, AbbVie, GlaxoSmith Kline, Merck, and Shire, Consultant of: AbbVie, Janssen, Pfizer, Amgen, Takeda, and Gilead, C. Lu Consultant of: Abbvie, Janssen, Ferring, and Takeda, Speakers bureau of: Janssen and Abbvie, C. Seow Consultant of: Advisory Boards: Janssen, Abbvie, Takeda, Ferring, Shire, Pfizer, Sandoz, Pharmascience, Fresenius Kabi, Amgen, Speakers bureau of: Janssen, Abbvie, Takeda, Ferring, Shire, Pfizer, Pharmascience, K. Novak Grant / Research support from: AbbVie and Janssen, Consultant of: Advisory board fees from AbbVie, Janssen, Pfizer, Ferring, and Takeda, speaker’s fees from AbbVie, Janssen, and Pfizer, R. Panaccione Consultant of: Abbott, AbbVie, Alimentiv (formerly Robarts), Amgen, Arena Pharmaceuticals, AstraZeneca, Biogen, Boehringer Ingelheim, Bristol-Myers Squibb, Celgene, Celltrion, Cosmos Pharmaceuticals, Eisai, Elan, Eli Lilly, Ferring, Galapagos, Fresenius Kabi, Genentech, Gilead Sciences, Glaxo-Smith Kline, JAMP Bio, Janssen, Merck, Mylan, Novartis, Oppilan Pharma, Organon, Pandion Pharma, Pendopharm, Pfizer, Progenity, Protagonist Therapeutics, Roche, Sandoz, Satisfai Health, Shire, Sublimity Therapeutics, Takeda Pharmaceuticals, Theravance Biopharma, Trellus, Viatris, UCB. Advisory Boards for: AbbVie, Alimentiv (formerly Robarts), Amgen, Arena Pharmaceuticals, AstraZeneca, Biogen, Boehringer Ingelheim, Bristol-Myers Squibb, Celgene, Eli Lilly, Ferring, Fresenius Kabi, Genentech, Gilead Sciences, Glaxo-Smith Kline, JAMP Bio, Janssen, Merck, Mylan, Novartis, Oppilan Pharma, Organon, Pandion Pharma, Pfizer, Progenity, Protagonist Therapeutics, Roche, SandozShire, Sublimity Therapeutics, Takeda Pharmaceuticals, Speakers bureau of: AbbVie, Amgen, Arena Pharmaceuticals, Bristol-Myers Squibb, Celgene, Eli Lilly, Ferring, Fresenius Kabi, Gilead Sciences, Janssen, Merck, Organon, Pfizer, Roche, Sandoz, Shire, Takeda Pharmaceuticals, C. Ma Grant / Research support from: Ferring, Pfizer, Consultant of: AbbVie, Alimentiv, American College of Gastroenterology, Amgen, AVIR Pharma Inc, BioJAMP, Bristol Myers Squibb, Celltrion, Ferring, Fresenius Kabi, Janssen, McKesson, Mylan, Sanofi/Regeneron, Takeda, Pendopharm, Pfizer, Roche, Speakers bureau of: : AbbVie, Amgen, AVIR Pharma Inc, Alimentiv, Bristol Myers Squibb, Ferring, Fresenius Kabi, Janssen, Takeda, Pendopharm, and Pfizer

